# Unraveling the gut microbiome of the long-lived naked mole-rat

**DOI:** 10.1038/s41598-017-10287-0

**Published:** 2017-08-29

**Authors:** Tewodros Debebe, Elena Biagi, Matteo Soverini, Susanne Holtze, Thomas Bernd Hildebrandt, Claudia Birkemeyer, Dereje Wyohannis, Alemayehu Lemma, Patrizia Brigidi, Vulk Savkovic, Brigitte König, Marco Candela, Gerd Birkenmeier

**Affiliations:** 1 Institute of Medical Microbiology, Faculty of Medicine, University of Leipzig, Liebigstrasse 21, 04103 Leipzig, Germany; 2 Department of Pharmacy and Biotechnology, Alma Mater Studiorum, University of Bologna, Bologna via Belmeloro 6, Bologna, 40126 Italy; 3 Department of Reproduction Management, Leibniz-Institute for Zoo and Wildlife Research, Alfred-Kowalke-Strasse 17, 10315 Berlin, Germany; 4 Institute of Analytical Chemistry, University of Leipzig, Linnestrasse 3, 04103 Leipzig, Germany; 5 College of Natural Sciences, Addis Ababa University, Addis Ababa, Ethiopia; 6 College of Veterinary Medicine and Agriculture, Addis Ababa University, Addis Ababa, Ethiopia; 7 Saxon Incubator for Clinical Translation, University of Leipzig, Phillip-Rosenthal-Str. 55, 04103 Leipzig, Germany; 8 Institute of Biochemistry, Faculty of Medicine, University of Leipzig, Johannisallee 30, 04103 Leipzig, Germany; 9 College of Medicine and Health Sciences, Bahir Dar University, Bahir Dar, Ethiopia

## Abstract

The naked mole-rat (*Heterocephalus glaber*) is a subterranean mouse-sized African mammal that shows astonishingly few age-related degenerative changes and seems to not be affected by cancer. These features make this wild rodent an excellent model to study the biology of healthy aging and longevity. Here we characterize for the first time the intestinal microbial ecosystem of the naked mole-rat in comparison to humans and other mammals, highlighting peculiarities related to the specific living environment, such as the enrichment in bacteria able to utilize soil sulfate as a terminal electron acceptor to sustain an anaerobic oxidative metabolism. Interestingly, some compositional gut microbiota peculiarities were also shared with human gut microbial ecosystems of centenarians and Hadza hunter-gatherers, considered as models of a healthy gut microbiome and of a homeostatic and highly adaptive gut microbiota-host relationship, respectively. In addition, we found an enrichment of short-chain fatty acids and carbohydrate degradation products in naked mole-rat compared to human samples. These data confirm the importance of the gut microbial ecosystem as an adaptive partner for the mammalian biology and health, independently of the host phylogeny.

## Introduction

The composition and functionality of complex and rich community of microbes living on the surfaces and cavities of the mammal’s body, i.e. microbiota, is well known to be crucial for the health maintenance of the host. An extremely rich and diverse microbial ecosystem inhabits the gastrointestinal tract collectively named as gut microbiota. Studies on humans have demonstrated that the gut microbiota strongly impacts on the prevention of disorders and pathologies, such as obesity and metabolic syndrome, cardiovascular diseases, inflammatory bowel diseases, as well as several types of cancer^1, 2^. The gut microbiota can indeed influence the education and homeostasis of the immune system and metabolism, as well as brain functionality, with unintelligible long-term effects on human health and lifespan^3, 4^.

The uniqueness of the different combinations of many impacting variables, such as perinatal events, lifestyle, diet, physiology, and clinical history, result in the outstanding individuality of the gut microbiota, not only in terms of phylogenetic composition, but also regarding the microbiota assembly and its fluctuations throughout the whole human life. In a mutualistic scenario these variations are the result of an adaptive process, aimed at the real-time optimization of the gut microbiome services in response to endogenous and exogenous variables^5^.

The impact of the gut microbiota on human health is a topic of huge interest for the scientific community, as demonstrated by the ever-increasing amount of studies on the microbiological peculiarities of the human gut ecosystem within the context of different lifestyles, genetic backgrounds or pathologies. Laboratory animal models, such as mice, rats and their germ-free counterparts, are commonly used to increase our biological comprehension of the microbiota-host interaction^6, 7^. However, despite their extraordinary relevance, laboratory models have intrinsic limitations, such as lack of the heterogeneity and the biological complexity of their wild counterpart. More recently, the gut microbiota of wild animals with peculiar dietary habits^8^ or physiological features^9, 10^ have become a topic of research interest, aiming at identifying the specialized and extreme host-microbiota alliances. These pioneer research work allowed to explore the biological limits of the gut microbiome capacity to complement mammalian biology and shedding light on the contribution of intestinal microbes to the host phenotype. Indeed, studying models of extreme adaptation, researchers hope to come to conclusions that could be translated to aspects of human health. For instance, studying the microbiota fluctuations during alternation of feeding, overfeeding and fasting in large hibernating animals can give insights into how to restore a healthy metabolism after a period of forced restricted (enteral) nutrition, or prolonged metabolic depression^11^.

It is a matter of fact that, by preserving the biological homeostasis of the human host, the gut microbiota has a role of primary importance in supporting human longevity^12^. However, only few hypotheses on the mechanisms involved have been advanced. Longevity is a tricky trait to be studied in humans, because it is a rare event, with an incredible amount of confounding genetic, lifestyle and clinical variables, both past and present. Still, the microbiota of human populations with extraordinary longevity rate is being investigated across geographical zones^13–15^ and interesting hypotheses on the role of the microbiome in health-maintenance during aging are being advanced.

In this scenario, the naked mole-rat (*Heterocephalus glaber*) might represent an extremely interesting model to study health and longevity, since, like for human beings, in naked mole rat the selection against aging is strongly reduced^16^. This eusocial, subterranean mouse-sized mammal, native to the arid and semi-arid regions of the Horn of Africa, occupies underground mazes of sealed tunnels and lives a very long life (30 years, approximately 8 times longer than common mice and rats) in large colonies with only one breeding queen and few breeding males^17^. Phylogenetically, this small mammal is classified within the newly-defined family Heterocephalidae, separated from the other African mole-rat species (Bathyergidae)^18^; both Heterocephalidae and Bathyergidae belong to the order Rodentia suborder Hystricognathi, together with other non-murine rodents such as the guinea pig, chinchilla and capybara. The naked mole-rat shows few age-related degenerative changes^19, 20^, displays an elevated tolerance to oxidative stress^21^, and its fibroblasts have shown resistance to heavy metals, DNA damaging agents, chemotherapeutics and other poisonous chemicals^22, 23^. Moreover, this mammals show remarkably small susceptibility to both spontaneous cancer and induced tumorigenesis^24–28^. These features of the naked mole-rat are maintained throughout their long lifespan, making this rodent a putative animal example of impressively prolonged “healthspan”^29^. Moreover, the within-colony low genetic diversity (possibly due to the high inbreeding rate)^30^, the climatologically stable underground habitats, and the constant diet (mainly tubers and other underground plant storage organs), make the naked mole-rat a unique model for studying the microbiota-host interaction, focusing on the ability of the gut microbes to contribute to health maintenance during aging^31^.

Here, we characterized the gut microbiota of the naked mole-rat by next generation sequencing based on the 16 S rRNA gene regions in comparison to different mammals, aiming at understanding of how the rodent´s gut microbiota profile aligns with human microbiome and that of other mammals. Furthermore, we studied the gut metabolome by GC-MS. Altogether; these data will possibly give new insights into the link between microbiota and a healthy longevity.

## Results and Discussion

The bacterial DNA extracted from the 35 naked mole-rat fecal samples was phylogenetically characterized by 16 S rRNA gene (V3-V4 region) Illumina sequencing. A total of 1,596,107 high-quality reads was obtained. The number of filtered sequences per sample ranged between 40,931 and 45,392 (mean 44,411). Reads were clustered in 15,863 operational taxonomic units (OTUs) at 97% of identity. At phylum-level the gut microbiota of the long-lived naked mole-rat is largely dominated by Firmicutes (average relative abundance (rel.ab.), 40.8%) and Bacteroidetes (38.8%), followed by Spirochaetes (12.0%), Actinobacteria (2.7%), Proteobacteria (2.6%), Synergistetes (1.3%) and other phyla at rel.ab. < 0.1% (Supplementary Fig. 1). The most represented families of Bacteroidetes in the naked mole-rat were assigned to *Prevotellaceae* (11%), *Paraprevotellaceae* (8.8%), an unclassified family of Bacteroidales (6.2%), *Porphyromonadaceae* (3.0%), and S24.7 lineage of Bacteroidetes (4.0%). The predominant family of the Firmicutes was *Lachnospiraceae* (17.6%), followed by an unclassified family of Clostridiales (6.1%), *Ruminococcaceae* (5.7%), *Veillonellacaea* (4.7%) and *Clostridiacaea* (4.1%) (Supplementary Fig. 2).

**Figure 1:**
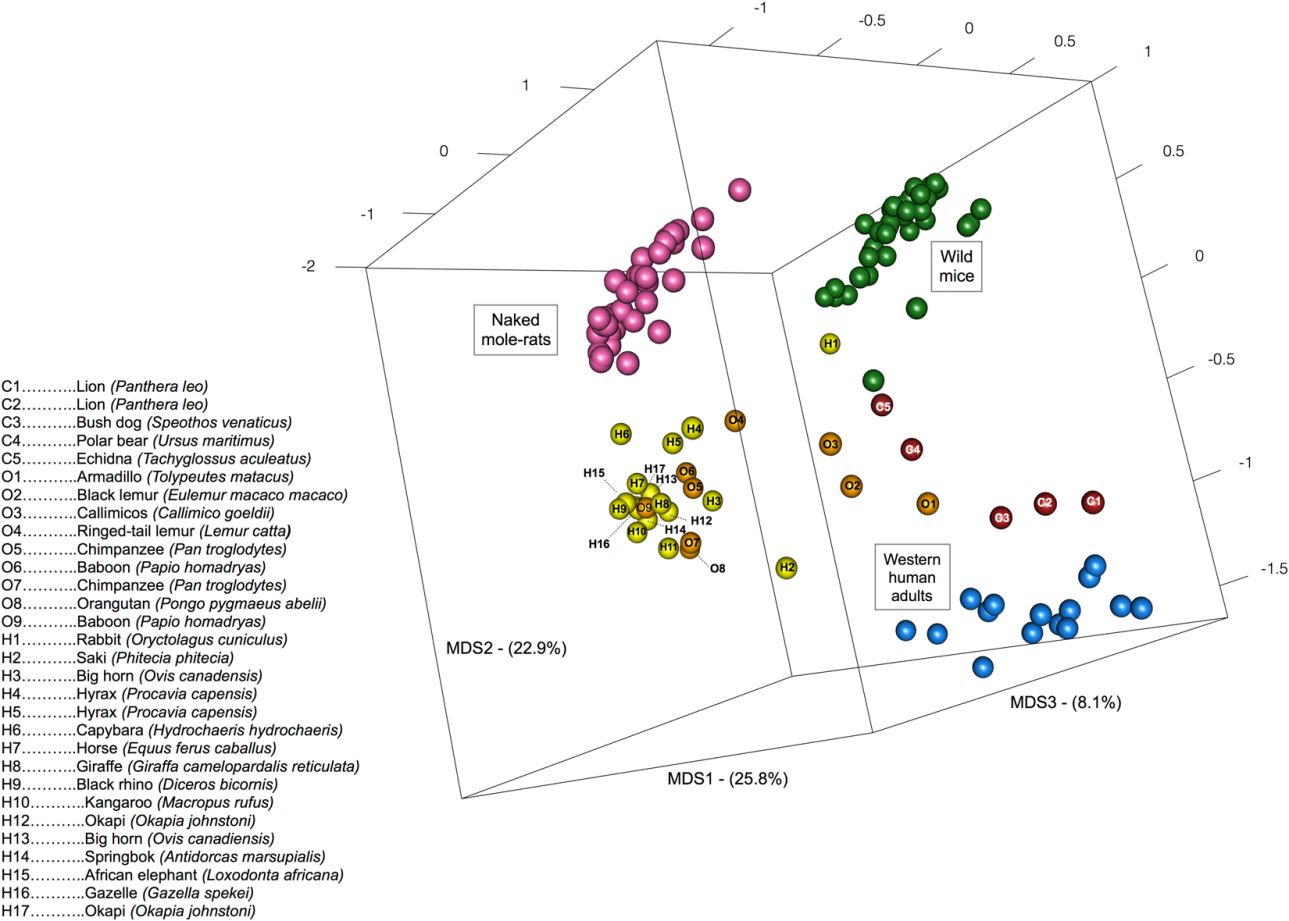
3D PCoA obtained by Bray-Curtis distance matrix showing the separation between naked mole-rats (pink), mice (green), western human adults (blue) and a group of different terrestrial mammalian species (carnivores in red (C1-C5), omnivores in orange (O1-O9), herbivores in yellow (H1-H17)) based on their gut microbial composition. Gut microbiota composition of terrestrial mammalian species was retrieved from Muegge *et al*.^34^, identification of these mammals is provided in the legend (left). Wild mouse gut microbiota was obtained from Weldon *et al*.^33^. First, second and third principal component are showed, accounting for 25.8%, 22.9% and 8.1% of the total variance in the dataset.

In order to obtain an ecological perspective on the naked mole-rat microbiota composition, the obtained profiles at genus-level were compared to that of humans^13, 32^, wild mice (*Mus musculus domesticus*)^33^ and other mammals^34^, in a PCoA based on Bray-Curtis distances between samples (Fig. 1). Naked mole-rat microbiota clustered separately from both mice and western humans, with the mixed mammals from the comprehensive study of Muegge *et al*.^34^ dispersed in between. The dispersion of the samples might have been influenced by the fact that the most of the animals in the study of Muegge *et al*.^34^ were kept captive in the same zoo environment, but it is still interesting to see that the naked mole-rat intestinal ecosystem emerged as a differently assembled microbiota. This could be linked to both the peculiar physiology and genetics of this rodent, and to the fact that it is the first completely subterranean mammal of which the microbiota have been studied. Interestingly, the closest animal sample to the naked mole-rat cluster belonged to the capybara (*Hydrochoerus hydrochaeris*), with which the naked mole-rat shares the suborder Hystricognathi. This confirmed the dominant influence of the mammalian phylogeny in determining the gut microbiota structure^35, 36^.

The Simpson diversity index calculated on the genus level profiles showed that the naked mole-rat microbiota was approximately as diverse as the human one (0.82 ± 0.03 and 0.84 ± 0.04, respectively) and significantly more diverse than that of wild mice (0.72 ± 0.14, Mann-Whitney test P < 0.001).

**Figure 2:**
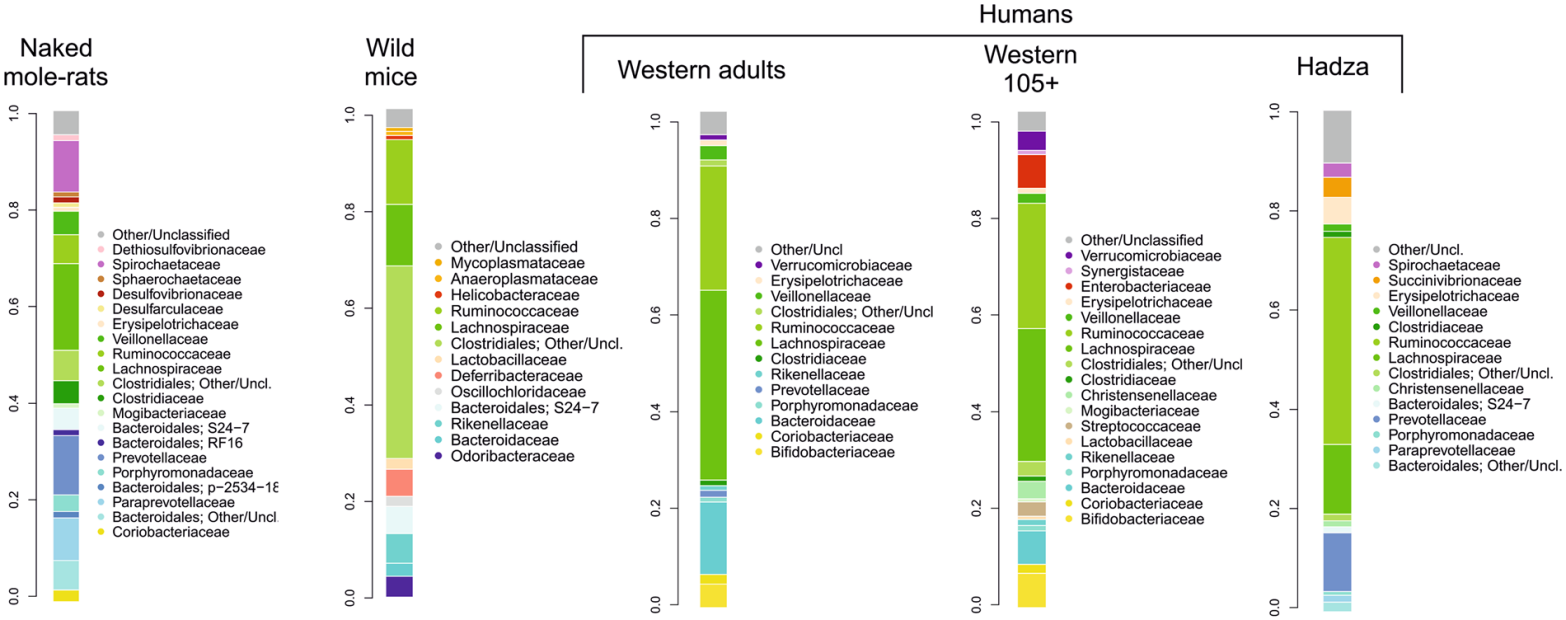
Family level gut microbiota average profiles of naked mole-rats, wild mice^33^, western human adults and supercentenarians^13^ and rural humans (Hadza)^32^. Families with average rel.ab > 0.8% are plotted. Color legends are reported for each profile to improve readability. Bacteroidetes and Firmicutes families are plotted in shades of blue and green, respectively.

The gut microbiome from wild mice, western healthy human adults (aged 22–48)^13^, African Hadza hunter-gatherers (aged 8–70)^32^ and western human supercentenarians (aged 105–109)^13^ were selected as models for a finest comparison with the naked mole-rat microbiome. While wild mice and western human adults were selected as representative of the most explored reference ecosystem for mammalians, microbiomes from supercentenarians and the Hadza hunter gatherers were chosen as representative of particularly successful microbiota-host mutualistic configurations, the first considered to support longevity^13^ and the second the host homeostasis in a complex environment^37^. In the context of this family-level comparative analysis (Fig. 2), with respect to wild mice and humans, the naked mole-rat microbiota showed an expanded relative contribution of families from the phylum Bacteroidetes, with a more pronounced inter-phylum diversity (6 families with rel.ab. > 0.8% vs 3 or 4 in wild mice and the three human populations). Interestingly, bacteria of the family *Bacteroidaceae*, i.e. the most abundant Bacteroidetes member both in western humans, was not represented in the naked mole-rat. On the contrary, their Bacteroidetes fraction was composed mostly by *Prevotellaceae*, *Paraprevotellaceae*, *Porphyromonadaceae* and the recently identified family S24–7. This peculiar configuration, with the exception of the S24–7, resembles the one observed in the human rural population Hadza^32^ (Fig. 2). Another trait that the naked mole-rat microbiota had in common with the Hadza one was the presence of *Spirochaetaceae* (10.9% and 2.8% in average, respectively), and in particular of the genus *Treponema* (Supplementary Fig. 3). This genus was represented in the naked mole-rat microbiota by a diversified population (763 OTUs, related to around 20 different *Treponema* species), with an average diversity of ten *Treponema* species per individual at > 0.01%, and three species at > 1%. Five species (*T*. *amylovorum*, *T*. *brennaborense*, *T*. *porcinum*, *T*. *succinifaciens*, *T*. *zuelzerae*) were found in all naked mole-rat samples at > 0.01% (never totally absent), with *T*. *porcinum* as the most frequently represented (>1%, average rel.ab. 5%, in all individuals). It is likely that *Treponema*, similar to the genus *Prevotella*, increases the ability of the naked mole-rat to digest and extract valuable nutrition from fibrous naturally occurring plants, of which both the naked mole-rat and the Hadza hunter-gatherers diet are enriched, since this genus includes proficient cellulose and xylan hydrolyzers. *Treponema* is indeed considered as an “old friend” and it is assumed that this taxa has been lost from human gut flora due to industrialization and modern lifestyle^32, 38^.

Curiously, another feature of the naked mole-rat microbiota, i.e. the presence of the family *Mogibacteriaceae* (rel.ab. 0.8 ± 0.4%), offered a parallel with the microbiota of extremely aged humans (i.e. supercentenarians (105 + years old) in which this family was retrieved in similar amounts (0.6%) (Fig. 2)^13^. Naked mole-rat and supercentenarians are both models of healthy aging and, even if possessing completely different microbial ecosystems, the sharing of this small peculiarity is an interesting finding, possibly worth exploring in the future.

Finally, the naked mole-rat microbiota showed appreciable abundance of bacterial families able to use sulfate, sulfite or other sulfur-containing molecules as terminal electron acceptor for fermentative and/or respiratory metabolism, such as *Desulfovibrionaceae* (average rel.ab. 1.2 ± 0.5%), *Desulfarculaceae* (0.9 ± 0.4%), and *Dethiosulfovibrionaceae* (1.2 ± 0.4%). In particular, bacteria of the family *Desulfarculaceae*, non-fermenting microbes that oxidize organic substrates completely to carbon dioxide^39^, have never been observed in the gut ecosystem of any animal^40^. The ecological role of these bacteria in the naked mole-rat gut is difficult to foresee, however, it is interesting to point out that the subsoil of the Rift Valley, in which these animals dig their tunnel and which they rarely leave, is enriched in sulfate^41^. We hypothesize that the soil provides the host with a terminal election acceptor to support an alternative and peculiar oxidative metabolism in the gut, which, based on sulfate, could represent a new mutualistic configuration of the gut microbiome-host transgenomic metabolism in the mammalian gut. Inferred metagenomics obtained by PiCRUST analysis and comparison between the KEGG pathways relative abundances in the naked mole-rat, western humans and mice, showed that the gut metagenome of the naked mole-rat was significantly enriched in pathways related to the *tryptophan metabolism* (naked mole-rat, 0.18% of the totality of KEGG pathways; wild mouse, 0.15% (Mann Whitney P = 0.003); western humans, 0.10% (P = 0.001), as well as *glycine*, *serine*, *and threonine metabolism* (naked mole-rat, 0.88%; wild mouse, 0.77% (P = 0.03); western humans, 0.82% (P < 0.0001)). Interestingly, tryptophan metabolites, as the indolic compounds indol-3-proprionic acid and indol-3-aldeide and nicotinic acid, represent emerging health promoting gut microbiome factors, which, equally to SCFA, can support the host metabolic and immune homeostasis^42^.

As for the energetic metabolism, the microbiota of the naked mole-rat seemed to show a more pronouncedly oxidative potential, with respect to carbohydrate utilization, since it was significantly enriched in pathways related to the *citrate cycle* (naked mole-rat, 0.66%; wild mouse, 0.53% (P = 0.0002); western human, 0.50% (P = 0.03), as well as to the *ubiquinone and other terpenoid-quinone biosynthesis* (naked mole-rat, 0.21%; wild mouse, 0.14% (P < 0.0001); western human, 0.08% (P = 0.004), which is a known electron carrier in oxidative phosphorylation, and *terpenoid backbone biosynthesis* (naked mole-rat, 0.69%; wild mouse, 0.47% (P < 0.0001); western human, 0.59% (P = 0.02). These peculiarities seem to confirm that the microbiota of the naked mole-rat might show peculiarities in the metabolic layout, more shifted towards an anaerobic oxidative metabolism instead of fermentation, with respect to other previously studied mammals.

Among other pathways that were significantly enriched in the naked mole-rat microbiota were those related to the degradation of xenobiotics, such as toluene (naked mole-rat, 0.11%; wild mouse, 0.07% (P < 0.0001); western human, 0.05% (P = 0.001). This observation suggests a possible gut microbiome contribute to the tolerance of the naked mole-rat to poisonous compounds, that have been hypothesized by previous literature based on naked mole-rat cell lines^22, 23^.

**Figure 3:**
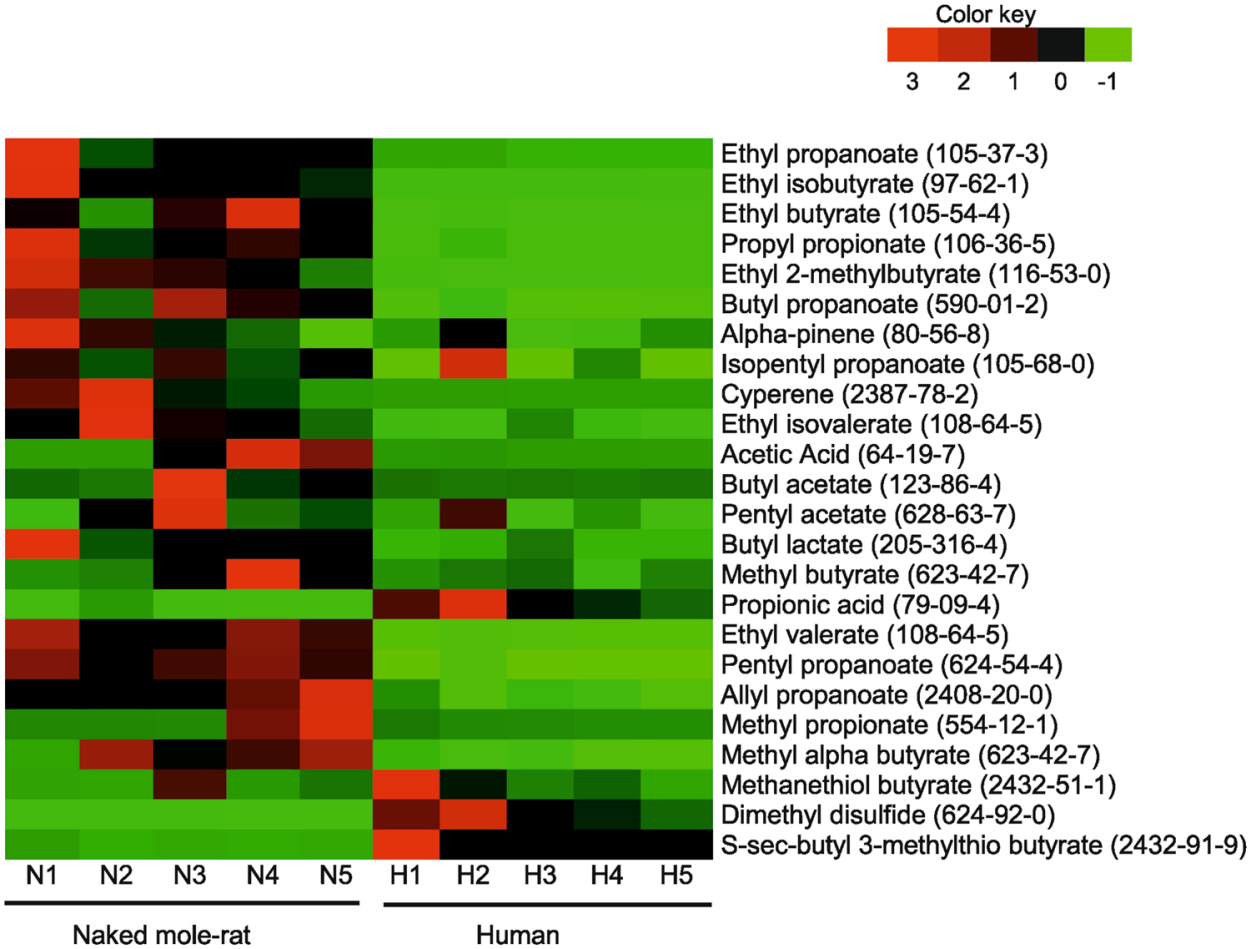
Heat map analysis of fecal metabolites from human and naked mole-rat. Individual relative abundance of each metabolite from naked mole-rat and human samples. Each column represents one sample (N = naked mole-rat; H = human). Red color represents a higher relative abundance, while green color illustrates a lower relative abundance.

The gut microbiota also play a pivotal role in the production of metabolites upon food fermentation, which have an important impact in maintaining gut health and construction of metabolic signal networks that are involved in the homeostasis of intestinal mucosa^43^. This prompted us to further analyze the volatile and polar gut metabolites of the naked mole-rat in comparison with humans. Indeed, even if the two mammals have different diet consumption patterns, the comparison of the respective metabolomes can help to provide information on the specific gut microbiome-dependent imprint on the host physiology. Interestingly, despite the limited sample size of our metabolome study, a higher relative abundance of volatile metabolites of food fermentation products was observed in the naked mole-rat compared with human (Fig. 3), indicating that the naked mole-rat microbiota is also appropriately equipped with a fermentative fraction that enable the rodents’ adaptation to a wide range of plant-originated diet^31^. These metabolic pathways culminate in the production and release of the major short-chain fatty acids (SCFA) such as acetate, propionate and butyrate, phytochemicals that were abundant in naked mole-rats compared to humans.

Notably, several mechanisms have been discussed to explain the cancer resistance of the naked mole-rat^25–27^. In many cancers inflammatory conditions precede the development of malignancy and smoldering inflammation aids proliferation and survival of malignant cells, stimulate angiogenesis and metastasis and subvert adaptive immunity^44^. Indeed, microbiota–derived SCFAs and their esters have remarkable colonic health-promoting and antineoplastic properties. They are the preferred energy source for colonocytes, maintain mucosal integrity and suppress inflammation and carcinogenesis through effects on immunity, gene expression and epigenetic modulation^45^. Most importantly, butyrate improves intestinal epithelial cells junctional integrity which prevents bacterial and toxin penetration into remote organs of the host^46^.

**Figure 4:**
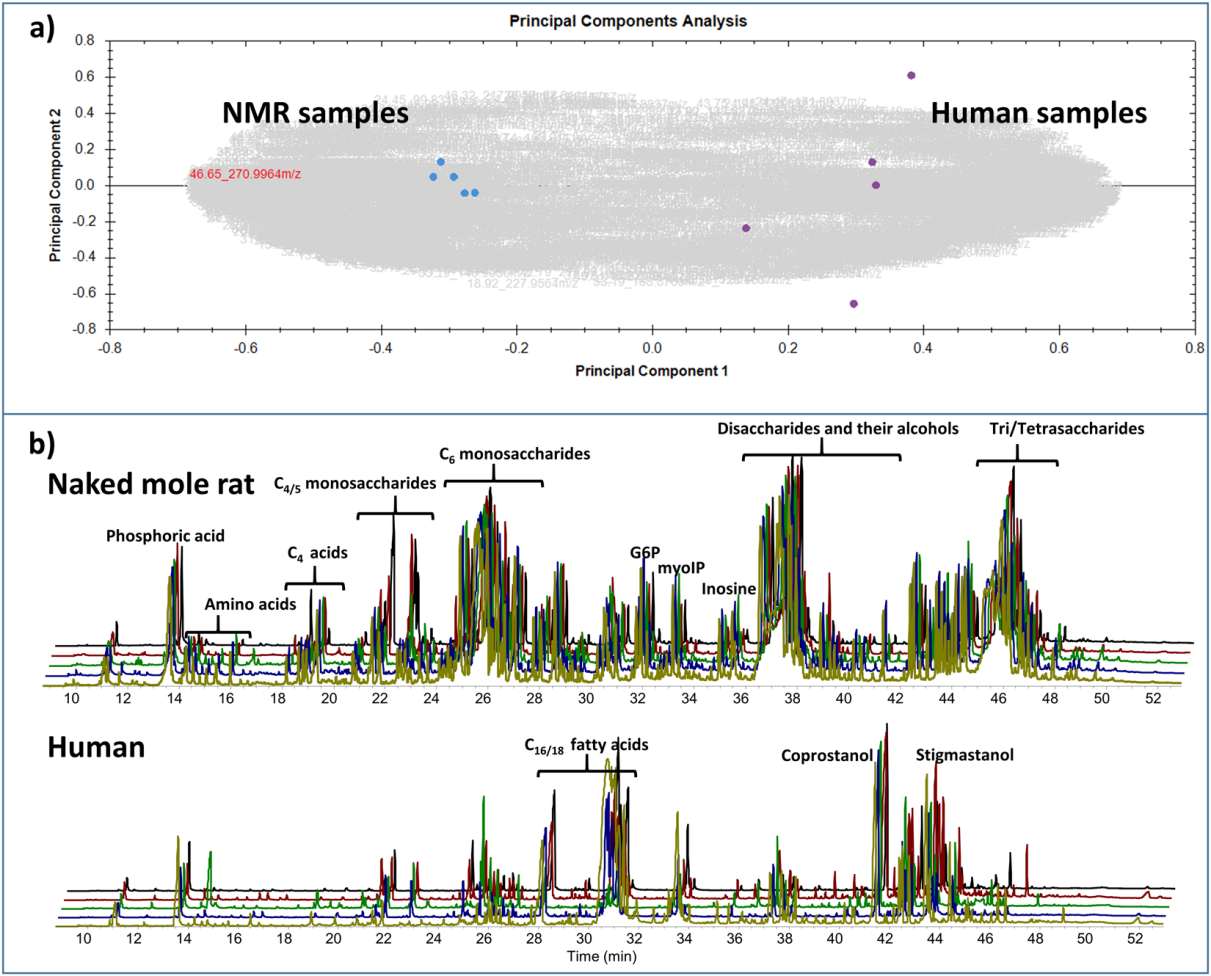
Polar metabolites in feces of the naked mole-rat. (**a**) Principal components analysis of GC-MS analysis of polar metabolites from the feces of naked mole-rat (NMR) and human samples (n = 5 each). The more distant the samples are in this graphic, the more their compound pattern differs. Naked mole-rat and human samples are separated on the level of principal component 1 which represents 58% of the variance; principle component 2 (10% variance) represents the variance within the replicates of one group. Clearly, naked mole-rat samples are far more distant to human samples than to each other. (**b**) Total ion current of GC-MS analyses of polar metabolites from the feces samples of naked mole-rat and human (n = 5 each). Top: Naked mole-rat replicates; Bottom: human sample replicates. Compound groups are labeled using brackets.

The enrichment of bacteria involved in carbohydrate metabolism as shown above is corroborated by GC-MS analysis of polar metabolites in the gut (Fig. 4). In contrast to human samples which are dominated by fatty acids and steroids including coprostanol, the naked mole-rat samples displayed abundancy of sugar compounds including mono, di- and oligosaccharides and their alcohols including amino- and small organic acids. It is reasonable to assume that this may reflect their predominant plant-derived dietary pattern^31^. Recently, it was found that naked mole-rats can survive O_2_ derivation (anoxia) up to 18 minutes^47^. This is obviously due to increased fructolysis generating fructose-1-phosphate to fuel the glycolytic metabolism in brain and heart under near-anaerobic conditions. Increased blood levels of fructose and the disaccharide sucrose were detected in naked mole-rat during anoxia. According to our results, we believe that the intestine of the naked mole-rat may function as a huge reservoir for such mono- and disaccharides. Fructose can enter cells via GLUT2 and GLUT5. GLUT5 is highly expressed in the intestine, kidney, liver, heart and brain in naked mole-rat compared with mouse^47^. Therefore, it is feasible to assume that under control of hypoxia/anoxia, these transporters might channel the sugars from the intestine into blood in short time.

Taken together, our data shows that the gut microbiota of the naked mole-rat not only shows peculiarities related to the specific living environment, such as the enrichment in bacteria able to metabolize sulfate, but it is also well equipped to obtain a relatively high abundance of a wide range of health-promoting metabolites from the possibly limited diversity of dietary substrates which might be available in their subterranean living environment.

## Conclusions

The naked mole-rat possesses a unique gut microbiome composition, which is the result of the host phylogeny and its peculiar ecology. This microbiome layout has many compositional and functional peculiarities - such as the propensity for an oxidative metabolism, an enhanced capacity to produce SCFA and mono- and disaccharides, as well as the peculiar structure within Bacteroidetes, the high load and diversity of Spirochetaceae and the presence of Mogibacteriaceae - some of which are shared with gut microbial ecosystems considered as models of healthy aging, as well as metabolic and immune homeostasis. This might suggest a possible role of the gut microbiota as a universal contributor to mammalian health, which goes beyond the host phylogeny and ecology constrains, supporting health and longevity of the mammalian host.

Moreover, even if confirmatory functional studies need to be carried out, our findings seem to suggest a capacity of the naked mole-rat gut microbiota to utilize soil sulfate as a terminal electron acceptor to sustain an anaerobic oxidative metabolism in the gut. This could represent an unprecedented ecological equilibrium. Specific for subterranean animals, this sulfate-dependent metabolism may further highlight the importance of the gut microbial ecosystem as an adaptive partner for the mammalian biology, which exerted a strategic role in the mammalian co-evolutionary processes.

Furthermore, the observed abundance of mono- and disaccharides in the intestine of naked mole-rat and their relation to the anoxia-resistance could be avail of managing the outcome of hypoxic situations in human.

## Material and Methods

### Sample collection and storage

Study subjects were captured and detained from the Rift Valley ecosystem in the eastern part of Ethiopia. Briefly, the fecal samples from each animal were collected and immediately frozen in a liquid nitrogen tank and transported to Leipzig, Germany, and stored at −80 °C prior to further analysis. The study was approved and permitted by Ethiopian Wild Life and Agricultural Authorities (reference number 31/25/08 dated on 19^th^ November, 2015). Subject collection and sampling were performed in accordance with the Ethiopian Wild Life Law guideline and regulation. For the human gut metabolites study, fecal samples were obtained from five healthy western human volunteers (aged 25–35) who consumed a normal diet (no vegetarian, no vegan). Samples were stored at −80 °C prior to further analysis. Written informed consent was also obtained from all subjects and the study was approved by the local ethics committee of the Faculty of Medicine, University of Leipzig in accordance to the ICH-GCP guidelines (Reference No. 057–2010–08032010).

### DNA extraction

Microbial genomic DNA from fecal material was extracted by bead-beating technique. Briefly, one 3.5 mm glass bead, 0.2 g of 0.1 mm zirconia beads and 0.2 g of 1.0 mm zirconia beads (BioSpec Products, Thistle Scientific, Uddingston, Glasgow, UK) were added into a 2 mL screw cap tubes (Sarstedt, Nuernbrecht, Germany) and autoclaved. Thereafter, 1 mL of lysis buffer (500 mM NaCl, 50 mM Tris-HCl pH 8, 50 mM EDTA, 4% SDS) was added and approximately 250 mg of feces was suspended in this buffer. The samples were treated in MagNa Lyser Instrument (Roche Diagnostic, Manheim, Germany) at 6000 speed, three times for 1 min and samples were cooled for 30 s on ice between each treatment. Then, samples were kept at 95 °C for 15 min and then centrifuged at 4 °C for 5 min at full speed to pellet stool particles. Supernatants were harvested and 347 µL of 7.5 M ammonium acetate (Carl Roth GmbH & Co., Karlsruhe, Germany) was added, followed by incubation on ice for 5 min and centrifugation at full speed for 10 min. One volume of 2-propanol (Sigma-Aldrich, Taufkirchen, Germany) was added to each supernatant and incubated on ice for 30 min. The precipitated nucleic acids were collected by centrifugation at full speed for 15 min and washed with 70% ethanol. Pellets were suspended in 100 µL TE buffer (10 mM Tris-HCl pH 8, 1 mM EDTA, pH 8) and treated with 2 µL of DNase-free RNase (10 mg/mL) (Thermo Fischer Scientific, Darmstadt, Germany) at 37 °C for 15 min. Samples were further treated by 15 µL Proteinase K and DNA purification with DNeasy Blood and Tissue Kit (QIAGEN, Hilden, Germany) following the kit protocol. Finally, DNA concentration was determined by NanoDrop 1000 (Thermo Fisher Scientific) and the quality and integrity of DNA were checked by agarose gel electrophoresis (0.8% w/v).

### 16 S rRNA gene amplification and MiSeq Illumina sequencing

The 16 S rRNA gene PCR amplification and sequencing were performed as described by Biagi *et al*.^13^. Briefly, the V3-V4 region of the 16 S rRNA gene was PCR amplified in 50 μL volumes containing 25 ng of microbial DNA, 2X KAPA HiFi HotStart ReadyMix (KAPA Biosystems, Resnova, Rome, Italy), and 200 nmol/L of S-D-Bact- 0341-b-S-17/S-D-Bact-0785-a-A-21 primers carrying Illumina overhang adapter sequences^48^. Thermal cycle consisted of 3 min at 95 °C, 25 cycles of 30 s at 95 °C, 30 s at 55 °C, and 30 s at 72 °C, and a final 5-min step at 72 °C. The 460 bp amplicons were purified with a magnetic bead-based clean-up system (Agencourt AMPure XP; Beckman Coulter, Brea, CA, USA) and sequenced on Illumina MiSeq platform using a 2 × 300 bp paired end protocol, according to the manufacturer’s instructions (Illumina, San Diego, CA, USA). Briefly, an indexed library for each sample was prepared by limited-cycle PCR using Nextera technology and further cleaned up with AMPure XP magnetic beads (Beckman Coulter). Libraries were pooled at equimolar concentrations (4 nM), denatured and diluted to 6 pmol/L before loading onto the MiSeq flow cell.

### GC-MS analysis of feces composition

Approximately 250 mg of fecal samples were suspended in 1 mL distilled water in 10 mL headspace glass vials and thoroughly mixed by vortexing. Afterwards, the volatile organic compounds (VOC) were analyzed by gas chromatography coupled with electron-impact ionization mass spectrometry (GC-MS) (GCMS-QP2010 Ultra System, Shimadzu, Kyoto, Japan). Finally, sample spectra were searched against the NIST 14 mass spectra library (National Institute of Standards and Technology, Gaithersburg, USA) to identify the detected metabolites. For polar metabolite analysis 50 mg of fecal sample was freeze-dried and subjected to GC-MS analysis as described by Hutschenrheuter *et al*.^49^. Automated peak picking, data alignment and statistical analysis (principal component analysis) was carried out using Progenesis QI 2.3 (nonlinear dynamics, WatersCorp, Durham USA).

### Data analysis and bioinformatics

Raw sequences were processed using a pipeline combining PANDAseq^50^. and QIIME^51^. Sequencing reads were deposited in the National Center for Biotechnology Information Sequence Read Archive (NCBI SRA; BioProject ID).

High-quality reads were binned into operational taxonomic units (OTUs) according the taxonomic threshold of 97% using UCLUST^52^. Chimera filtering was performed by discarding all singleton OTUs. Taxonomy was assigned using the RDP classifier against Greengenes database (May 2013 release) and relative abundances at different phylogenetic levels were calculated. Alpha rarefaction was analyzed by using Chao1, PD whole tree, observed species, and Shannon index metrics in order to verify the saturation of the sequencing method.

For comparison with the microbiota of other species and human populations, publicly available dataset of row sequences from the studies of Muegge *et al*.^34^, Weldon *et al*.^33^, Schnorr *et al*.^32^, and Biagi *et al*.^13^, were downloaded, OTU assignment was performed as above and genus-level relative abundances were calculated. Bray-Curtis distances were computed based on genus-level profiles using R software (https://www.r-project.org/) and the libraries vegan and stats. Principal Components Analysis (PCoA) was performed and a 3D graphical representation was obtained by using the R package rgl. Biodiversity of samples was quantified by computing Simpson diversity index using the function “diversity” of the R package vegan and the genus-level relative abundances for each considered samples.

Metagenome imputation of Greengenes-picked OTU was performed using PICRUSt (Phylogenetic Investigation of Communities by Reconstruction of Unobserved States)^53^ with default settings. The KEGG (Kyoto Encyclopedia of Genes and Genomes) Onthology (KO) database^54^ was used for functional annotation. Mann-Whitney U test was used to assess for significant differences between naked mole-rat, mouse and human imputed metagenome profiles. The p-values were corrected for multiple comparisons using the Bonferroni method. Corrected p < 0.05 was considered as statistically significant.

The difference of the relative abundance of metabolites was huge so that we normalized the data to the Z-score based on the following calculation ((*x−µ*)/*σ*), where *x* is the area value, *µ* is the mean area value over the whole dataset and *σ* represents the standard deviation.

## Electronic supplementary material

Supplementary Information
