# Supplementary material for: Unraveling the gut microbiome of the long-lived naked mole-rat
Source: Sci Rep. 2017 Aug 29;7:9590. doi: 10.1038/s41598-017-10287-0 (PMC5575099; doi:10.1038/s41598-017-10287-0)

Unraveling the gut microbiome of the long-lived naked mole-rat

Tewodros Debebe, Elena Biagi, Matteo Soverini, Susanne Holtze, Thomas Bernd Hildebrandt, Claudia Birkemeyer, Dereje Wyohannis, Alemayehu Lemma, Patrizia Brigidi, Vulk Savkovic, Brigitte König, Marco Candela, Gerd Birkenmeier

Supplementary files

Supplementary Figure 1 – Phylum level individual profiles (barplot) and average profile (pie chart) of naked mole-rat microbiota.

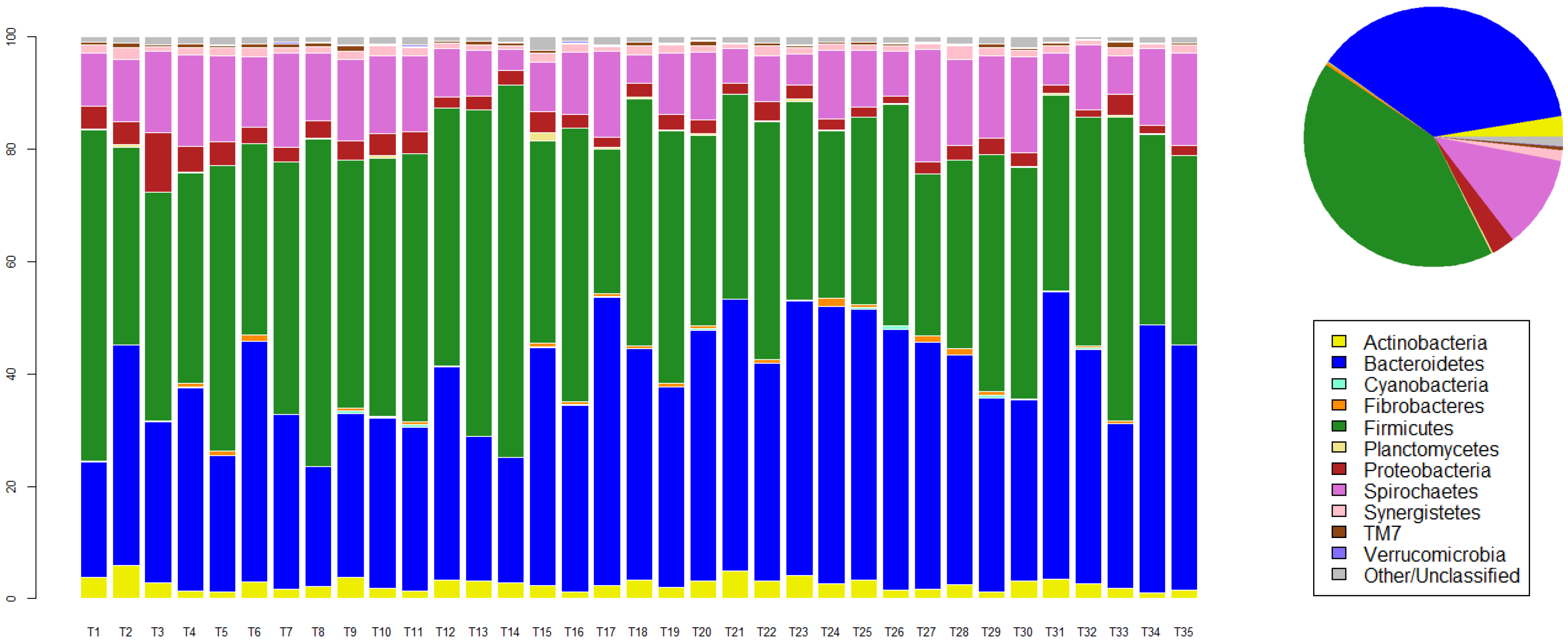

Supplementary Figure 2 – Family level individual profiles (barplot) and average profile (pie chart) of naked mole-rat microbiota.

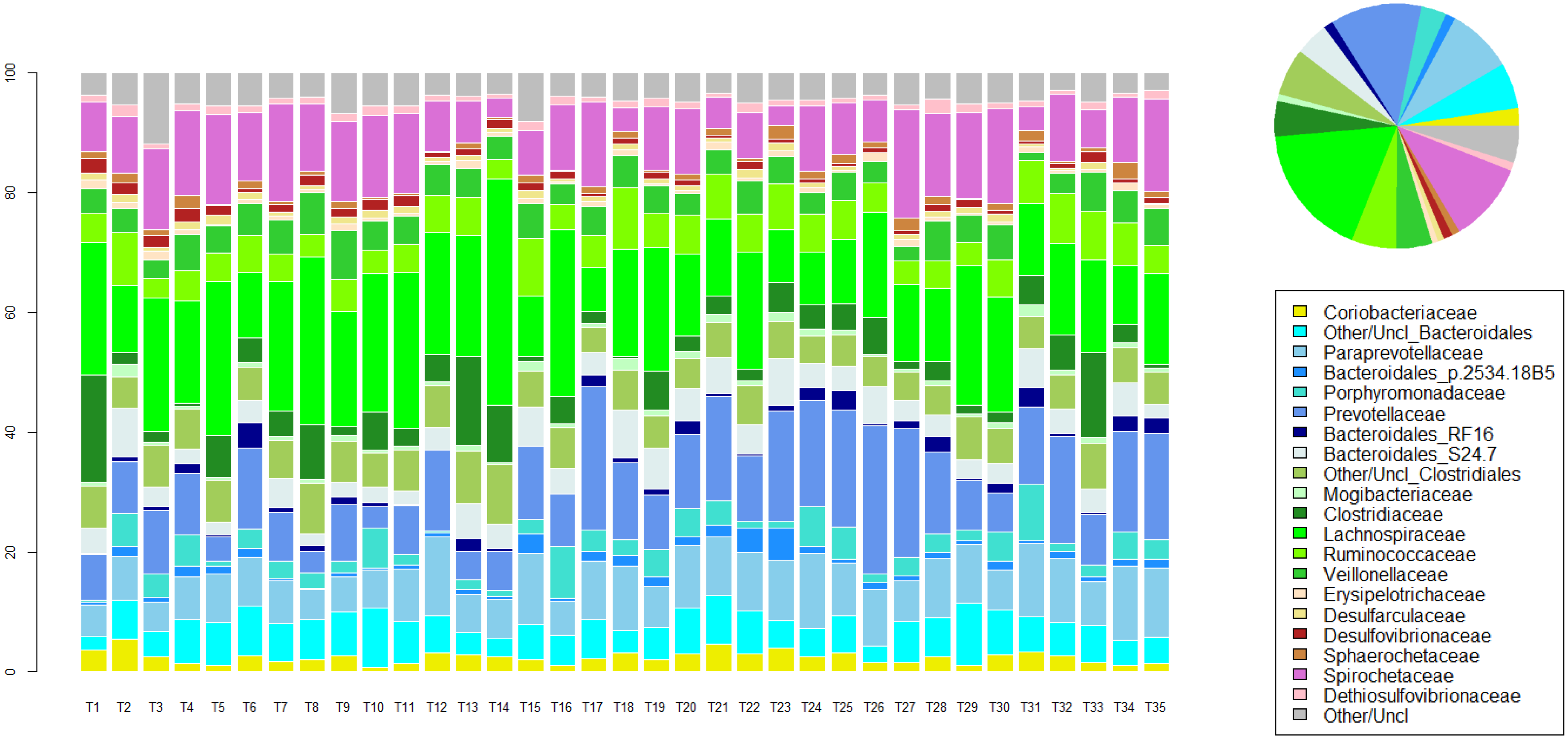

Supplementary Figure 3 – Genus level individual profiles (barplot) and average profile (pie chart) of naked mole-rat microbiota.

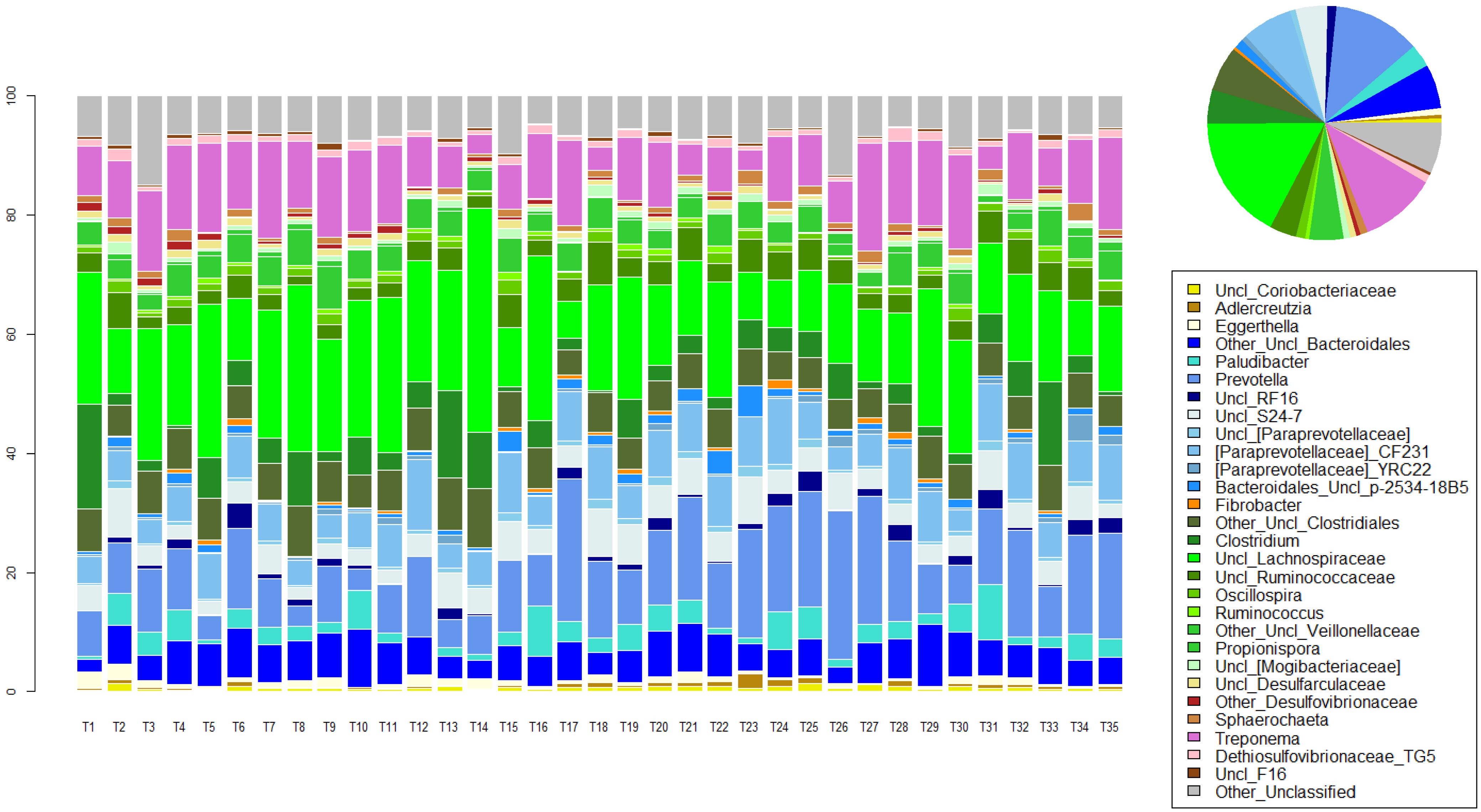

Supplement: Supplementary file 1 — Supplementary Information [file 41598_2017_10287_MOESM1_ESM.pdf]
